# Providing new formulation for white compound chocolate based on mixture of soy flour, sesame paste, and emulsifier: An optimization study using response surface methodology

**DOI:** 10.1002/fsn3.2111

**Published:** 2021-01-10

**Authors:** Abdul Fateh Hosseini, Mostafa Mazaheri‐Tehrani, Samira Yeganehzad, Seyed Mohammad Ali Razavi

**Affiliations:** ^1^ Department of Food Science and Technology Ferdowsi University of Mashhad (FUM) Mashhad Iran; ^2^ Department of Food Processing Research Institute of Food Science and Technology (RIFST) Mashhad Iran

**Keywords:** emulsifier, sesame paste, soy flour, textural properties, thermal properties, white compound chocolate

## Abstract

This study was aimed at evaluating the suitability of sesame paste as an ingredient in white compound chocolate using response surface methodology. A D‐optimal combined mixture‐process design with three mixture components, sesame paste (15%–30% w/w), soy flour (0%–15% w/w), and milk powder (0%–15% w/w) under variable amount of emulsifier was used to optimize textural (hardness, cohesiveness, and adhesive force) and thermal (*T*_onset_ and *T*_m_) properties of white compound chocolate containing sesame paste. The results showed that the linear effect of all the mixture components was significant (*p* < .05) on the responses. Applying a desirability function method, the optimum proportion of mixture components, and emulsifier level were as follows: sesame paste 15.5% w/w, milk powder 7.5% w/w, soy four 7% w/w, and emulsifier 0% w/w, respectively. At optimum point, hardness, cohesiveness, adhesive force, *T*_onset_, and *T*_m_ were 367.07 (N), 0.63, 8.46 (N), 28.1 (°C), and 33.7 (°C), respectively. The predicted values were confirmed through validation experiment.

## Graphical abstract

This study was aimed at evaluating the suitability of sesame paste as an ingredient in white compound chocolate using response surface methodology. A D‐optimal combined mixture‐process design with three mixture components, sesame paste (15%–30% w/w), soy flour (0%–15% w/w), and milk powder (0%–15% w/w) under the variable amount of emulsifier was used to optimize textural (hardness, cohesiveness, and adhesive force) and thermal (*T*_onset_ and *T*_m_) properties of white compound chocolate containing sesame paste.

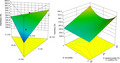

## 1 INTRODUCTION

Sesame paste, which is known as Ardeh in Iran, is obtained by milling dehulled roasted sesame seeds without any additives (Abu‐Jdayil, 2004; Lokumcu Altay & Ak, 2005). In terms of the chemical composition, it contains high amount of lipids (57–65 wt.%), proteins (23–27 wt.%), carbohydrates (6.4–9 wt.%), niacin (4.5 mg/100 g), thiamin (1.08 mg/100 g), and some minerals (Akbulut & Oklar, 2008). The notable nutritive value of sesame paste can be attributed to its proteins (rich in sulfur‐containing amino acids such as methionine and tryptophan), lipids (highly stable, with oleic and linoleic acids constituting most of the fatty acids), and important minerals and vitamins (Namiki, 1995). The consumption of sesame paste is very low compared with its country's total production. One of the reasons is that sedimentation of solid particles and oil separation causes the product to become two‐phase over time (Çiftçi et al., 2008). This unpleasant appearance leads to low consumer acceptance. Therefore, in order to increase the home use of sesame paste, it is important that investigation should be carried out to develop a compound chocolate containing sesame paste that will be attractive to the consumers because of its appealing taste, texture, and eating pleasure. Chocolate in general, however, is found to be low in protein (Pandey & Singh, 2011). Problem of low protein content in chocolate compound can be solved by incorporating soy flour. Therefore, formulation of white compound chocolate containing sesame paste and enriched with soy flour leads to a nutritional natural novel product. Several different types of vegetable fat can be used in place of cocoa butter to produce a compound chocolate, and it is more usual to find a cocoa butter substitute (CBS) in the manufacture of compound chocolates. CBS fats have a sharp melting point, giving similar texture properties to those of chocolate (Dale, 2017). Recently, many studies have reported the rheological properties of sesame paste and blends containing sesame paste. The details of the afore‐mentioned studies can be found in Alpaslan and Hayta (2002), Abu‐Jdayil (2004). Studies on oil separation problem in halva (a sesame paste product) are very limited (Elleuch et al., 2014; Ereifej et al., 2005; Guneser & Zorba, 2014). To our knowledge, no data have been published on the presence of the sesame paste and soy flour in white compound chocolate formulation. The objective of this study was optimizing the formulation of a white compound chocolate containing sesame paste, soy flour, milk powder, and emulsifier based on textural and thermal properties using a combined mixture‐process experimental design.

## 2 MATERIAL AND METHOD

### 2.1 Materials

The raw materials used in the production of white compound chocolate containing sesame paste are the following: sugar (Iran sugar Co.), cocoa butter substitution (composed of hydrogenated palm kernel oil, supplied from Indonesia, Fuji oil Inc.), skim milk powder containing 39.28% protein and 3.5% moisture (Golshad), heat‐processed whole soybean flour containing 22.08% oil and 39.28% protein (Toos Soyan), sesame paste (fat 50.64%, protein 21.87%, Simorgh Company), and monoglyceride (E471) (Danisco Company).

### 2.2 Chocolate production

All the white compound chocolate containing sesame paste (500 g batch for each formulation) was produced in laboratory ball mill. A weighed amount of CBS was melted in a microwave oven at a temperature of approximately 45–50°C. Suspension was prepared at ambient temperature (20–22°C) by adding soy flour, sesame paste, and milk powder to CBS while mixing evenly with a spatula. The suspension then was transferred to the ball mill (ball diameter 9.1 mm; mixer rotation speed 100 rpm). The applied refining time in the mill was 70 min at 65°C. During the first 10 min of the experiment, the icing sugar was gradually added. To obtain the desired flow characteristics, monoglyceride was added 15 min prior to the end of the process. After the established refining time, the refined suspension white compound chocolate was discharged into molds shaken gently to remove air bubbles and then placed in refrigerator at temperature of 10°C. After cooling and demolding, samples were wrapped in aluminum foil for packaging and conditioned at ambient temperature (20–22°C) for 24 hr prior to analysis.

### 2.3 Texture analysis

The texture profile analysis (TPA) with 30% compression was conducted on prepared samples using a texture analyzer (TA‐XT plus, Stable Microsystems Ltd.). Test settings were as follows: test speed of 1 mm/s, relaxation time of 5 s, and force of 20 g. Dimensions of the white compound chocolate containing sesame paste bar were 5 mm × 38 mm × 25 mm. The measured parameters included hardness, cohesiveness, and adhesive force. Mean values from three replicate measurements were calculated (Andrae‐nightingale et al., 2009).

### 2.4 Thermal properties

The thermal properties of white compound chocolate containing sesame paste were determined utilizing a SPICO‐DSC‐100. Approximately, 17–20 mg of the sample was placed in hermetically sealed aluminum pans with an empty pan as a reference. Melting profile was recorded by heating at 10°C/min from 10 to 80°C. Following parameters were extracted from the melting curve:

Onset temperature (*T*_onset_)
Maximum temperature (*T*_m_)

Each sample was analyzed in triplicate and mean values reported (Kiumarsi et al., 2020).

### 2.5 Experimental design

D‐optimal combined mixture‐process design was used to investigate the effect of two factors; the first one is mixture components, including A: sesame paste (15%–30% w/w), B: milk powder (0%–15% w/w), C: soy flour (0%–15% w/w) with A + B + C = 30% w/w, and the second is D: emulsifier (0%–2% w/w) on five responses (hardness, cohesiveness, adhesive force, *T*_onset_, and *T*_m_). To independently investigate the effect of the monoglyceride emulsifier, we considered it as a process variable. Other components of white compound chocolate containing sesame paste were sugar and CBS. Twenty combinations of the variables were selected by experimental design as shown in Table 1. Another control sample was included to compare the results to a commercial sample. All experiments were conducted at three replicates.

**TABLE 1:** Mixture optimal design matrix with uncoded values of the factors and observed textural and thermal responses

Run	Mixture components (% w/w)			Process variable (% w/w)	Characterization				
	Sesame paste (A)	Milk powder (B)	Soy four (C)	Emulsifier (D)	Hardness (N)	Cohesiveness	Adhesive force (N)	*T*_onset_ (°C)	*T*_m_ (°C)
1	15	7.5	7.5	0	332.448	0.61	8.3	29.3	34.1
2	15	0	15	1	288.401	0.55	6.24	26.7	32.2
3	30	0	0	0	234.213	0.51	8.16	27.4	32.2
4	15	7.5	7.5	1	330.857	0.6	6.85	26.6	32.5
5	22.5	7.5	0	2	246.262	0.5	7.73	27.3	33.3
6	22.5	0	7.5	2	263.483	0.47	6.63	25.6	32
7	15	15	0	1	317.641	0.64	7.83	29.6	34.1
8	30	0	0	0	246.523	0.51	7.6	27.7	32.6
9	22.5	0	7.5	0	276.868	0.55	7.19	28.6	33.9
10	30	0	0	2	216.857	0.4	8.20	27.1	32.8
11	15	15	0	0	346.448	0.66	8.26	25.7	33.5
12	22.5	7.5	0	0	326.441	0.57	9.81	28	33.5
13	25	2.5	2.5	1	282.835	0.53	8.26	26.7	33
14	15	0	15	0	382.368	0.64	7.14	27.2	33.5
15	22.5	7.5	0	0	280.562	0.54	9.86	27.5	33.4
16	15	0	15	2	397.087	0.72	4.92	22.6	34.2
17	22.5	0	7.5	0	322.184	0.62	7.77	27.6	32.8
18	15	7.5	7.5	2	415.908	0.73	5.12	27.5	32.7
19	15	15	0	2	384.428	0.72	5.36	28	34.1
20	30	0	0	2	270.764	0.48	7.13	23.4	30.6
Commercial sample	—	—	—	—	442.25	0.72	4.32	31.32	34.52

### 2.6 Statistical analysis

Design‐Expert version 10.0.1 statistical software (Stat‐Ease Inc.) was used to analyze the data. For each response, different models and their suitability were evaluated, and significant terms in generated mathematical models were determined by analysis of variance (ANOVA). The significance was judged at 5% probability level. The validity of the selected models was assessed by the coefficient of determination, *R* ^2^, adjusted‐*R* ^2^ (adj‐*R* ^2^), and coefficient of variation (CV). *R* ^2^ is defined according to its magnitude which is the ratio of the explained variation to the total variation. A good mathematical fitting model should have a large *R* ^2^ (larger than 80%) and adj‐*R* ^2^. CV expresses standard deviation as a percentage of the mean. Generally, CV should not be greater than 10% and small values of CV represent a better precision and reliability of the conducted experiments (Karazhiyan et al., 2011).

### 2.7 Optimization and validation

The numerical optimization process using the Design‐Expert software was carried out to optimize multiresponses based on desired function methodology. To describe the desirability of responses, they were either maximized or minimized (to target the control sample characteristics) while the mixture components and process variable were kept in range. To test the adequacy of the models, additional experiments at the optimum levels obtained by RSM optimization were conducted and the experimental data were compared with the predicted ones.

## 3 RESULT AND DISCUSSION

### 3.1 Model fitting

According to data analysis, one of the two polynomial models, linear × linear, and linear × quadratic was selected to be the most appropriate model to analyze the responses. Table 2 illustrates analysis of variance for the fitting models. A significant lack of fit reflects the failure of the models to represent the data in the experimental design in which points were not included in the regression (Karazhiyan et al., 2011). The ANOVA depicted that lack of fit was not significant for all output responses at 95% confidence level, meaning that the models represented the data appropriately. To check the model validity, *R* ^2^, adj‐*R* ^2^, and CV were also calculated. According to Table 2, *R* ^2^ of the models are all higher than 80%, indicating a good fit between the regression model and the experimental values and nonsignificant terms have not been included in the model (Karazhiyan et al., 2011). The *R* ^2^ values were 0.83, 0.95, 0.89, 0.83, and 0.81 for hardness, cohesiveness, adhesive force, *T*_onset_, and *T*_m_, respectively (Table 2). The CV values were 8.39, 4.28, 6.68, 2.4, and 1.1 for hardness, cohesiveness, adhesive force, *T*_onset_, and *T*_m_, respectively. A CV lower than 10 shows that variation in the mean value is low and satisfactorily develop an adequate response model.

**TABLE 2:** Model statistics and adequacy of the models for hardness, cohesiveness, adhesive force, *T*_onset_, and *T*_m_ responses

Response	Model mixture × emulsifier	C.V %	*df*	*R* ^2^	Adjusted *R* ^2^	*SD*	*F*‐value	*p*‐value	Lack of fit *p*‐value
Hardness	Linear × quadratic	8.39	3	.83	.79	26.65	21.29	<.0001	.57
Cohesiveness	Linear × quadratic	4.23	6	.95	.92	0.025	29.89	<.0001	.81
Adhesive force	Linear × linear	6.68	4	.89	.86	0.49	30.43	<.0001	.5
*T*_onset_	Linear × linear	2.45	3	.83	.79	0.66	20.68	<.0001	.26
*T*_m_	Linear × linear	1.1	3	.81	.72	0.36	15.59	<.0001	.85

Note

*T*_onset_ = onset temperature; *T*_m_ = maximum temperature.

### 3.2 Response surface plots

The relationship between dependent and independent variables can be illustrated using three‐dimensional response surface graphs generated by the model. The data were generated through keeping one variable at its respective level and varying the other three within the experimental range.

### 3.3 Textural and thermal properties

**FIGURE 1:**
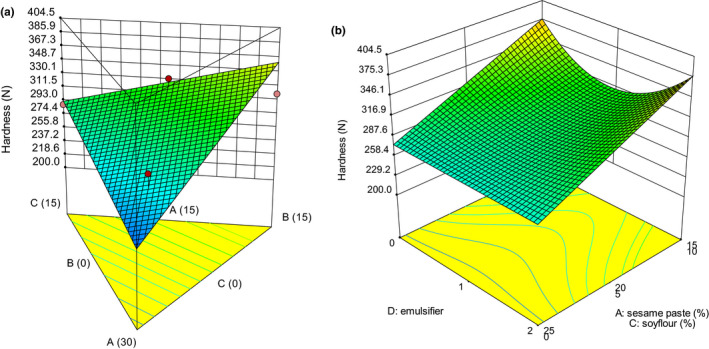
3‐D graphs corresponding to models fitted for hardness: (a) effect of mixture components (A: sesame paste, B: milk powder, and C: soy flour) while emulsifier is constant (1% w/w), (b) combined effects of emulsifier (D) and mixture components (A: sesame paste, C: soy flour) while milk powder is constant (5% w/w)

**FIGURE 2:**
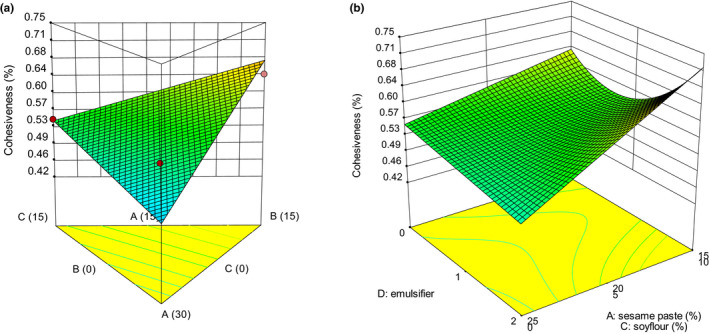
3‐D graphs corresponding to models fitted for cohesiveness: (a) effect of mixture components (A: sesame paste, B: milk powder, and C: soy flour) while emulsifier is constant (1% w/w), (b) combined effects of emulsifier (D) and mixture components (A: sesame paste, C: soy flour) while milk powder is constant (5% w/w)

**FIGURE 3:**
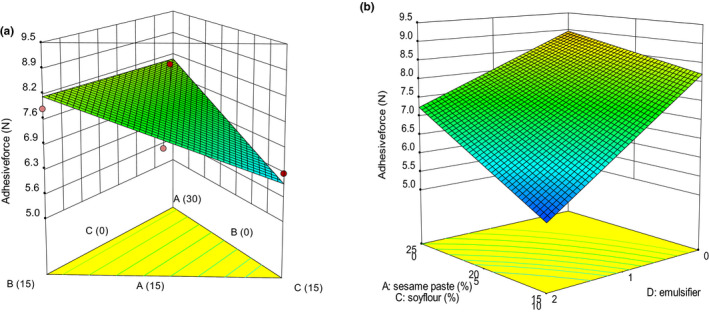
3‐D graphs corresponding to models fitted for adhesive force: (a) effect of mixture components (A: sesame paste, B: milk powder, and C: soy flour) while emulsifier is constant (1% w/w), (b) combined effects of emulsifier (D) and mixture components (A: sesame paste, C: soy flour) while milk powder is constant (5% w/w)

**FIGURE 4:**
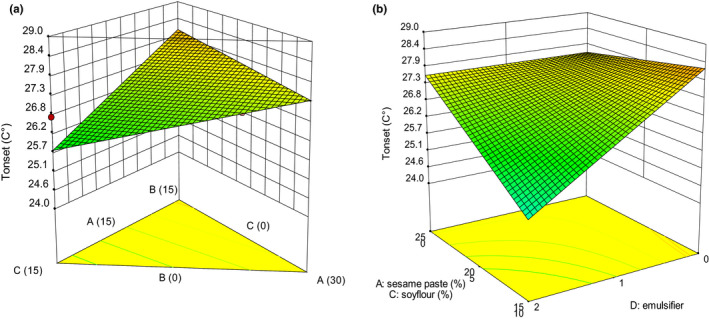
3‐D graphs corresponding to models fitted for *T*_onset_: (a) effect of mixture components (A: sesame paste, B: milk powder, and C: soy flour) while emulsifier is constant (1% w/w), (b) combined effects of emulsifier (D) and mixture components (A: sesame paste, C: soy flour) while milk powder is constant (5% w/w)

**FIGURE 5:**
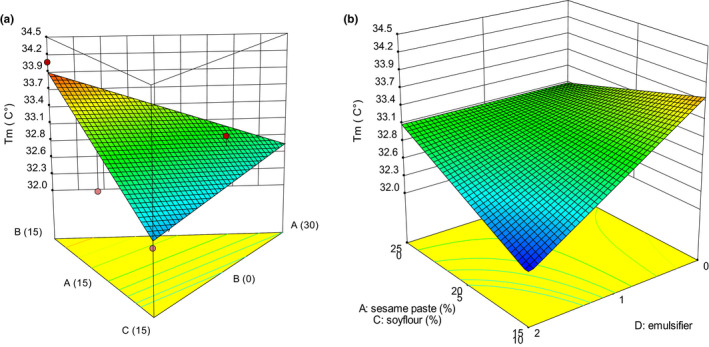
3‐D graphs corresponding to models fitted for *T*_m_: (a) effect of mixture components (A: sesame paste, B: milk powder, and C: soy flour) while emulsifier is constant (1% w/w), (b) combined effects of emulsifier (D) and mixture components (A: sesame paste, C: soy flour) while milk powder is constant (5% w/w)

Texture is one of the key attributes for consumers’ acceptance (Afoakwa et al., 2008b). Many textural properties can be used to describe the texture of chocolate, which includes hardness, cohesiveness, adhesiveness, gumminess, and chewiness (Glicerina et al., 2013). The variations of textural and thermal properties are presented in Figures 1, 2, 3, 4, 5. The values of *p* were used to evaluate the significance of every coefficient on the responses. The *p*‐values less than .05 show that model terms are significant.

The TPA hardness is defined as the peak force during the first compression cycle (Kahyaoglu et al., 2005). The hardness value had a linear (mixture components) × quadratic (emulsifier) model as illustrated in Table 3 where the linear effect of mixture components (A, B, and C) and the interaction between C and D (emulsifier) were significant (*p* < .05). The effect of mixture components and emulsifier on hardness are presented in Figure 1a,b. Increasing milk powder has a significant effect on hardness (Figure 1a). White compound chocolate formulations with a high amount of milk powder (15%) had the highest hardness, while the lowest hardness was achieved by a complete substitution of milk powder for sesame paste. As shown in the plot, the effect of sesame paste on the reduction of hardness was more pronounced than soy flour. According to Full et al. (1996), solid fat content (SFC) increases the chocolate hardness. It seems that the reduction in hardness is attributable to sesame paste and soy flour containing a high amount of vegetable oils, leading to a remarkable decrease in SFC and softening effect on white compound chocolate texture. In addition, the reduction in hardness was consistent with results of Ali et al. (2001) reported that in the blended fats, dissimilar fatty acid composition caused eutectic behavior which resulted in softened texture. Pandey and Singh (2011) stated that an increase in soybean oil decreased the chocolate hardness. It may be due to low melting point or low level of saturated fatty acids in soybean oil than cocoa butter.

**TABLE 3:** Polynomial mathematical models with interaction terms for different responses

Response	Equation
Hardness	+ 234.89 * A + 359.75 * B + 293.43 * C + 91.85 * CD^2^
Cohesiveness	+ 0.46 * A + 0.67 * B + 0.54 * C − 0.054 * AD + 0.036* BD + 0.046 * CD + 0.14 * CD^2^
Adhesive force	+ 8.08 * A + 8.15 * B + 5.98 * C − 2.58 * BD − 0.97 * CD
*T*_onset_	+ 27.36 * A + 28.1 * B + 25.74 * C − 2.61 * CD
*T*_m_	32.7 * A + 33.94 * B + 32.31 * C − 1.33 * CD

Note

Where A = sesame paste; B = milk powder; C = soy four; D = emulsifier; *T*_onset_ = onset temperature; *T*_m_ = maximum temperature.

The effect of emulsifier on hardness at a constant amount of milk powder (5% w/w) is given in Figure 1b. At high proportion of sesame paste, emulsifier did not have large effect on the hardness compared with lower proportion of sesame paste. It seemed that at high level of sesame paste due to the high amount of sesame oil, the effect of emulsifier was not observed well. At low proportion of sesame paste (15% w/w), increasing emulsifier up to a certain value (1% w/w) decreased the hardness but which then increased at increased value of emulsifier (2% w/w).

The TPA cohesiveness is defined as the ratio of the positive force area during the second compression (*A*_2_) to the force area of the first compression (*A*_1_; Messens et al., 2000). The linear (mixture components) × quadratic (emulsifier) model in Table 3 revealed that linear effect of mixture components (A, B, and C) and their interaction with D on cohesiveness were significant (*p* < .05). The effect of mixture components and emulsifier on cohesiveness are shown in Figure 2a,b. As shown by Figure 2a sides A–C and A–B, the increase in milk powder and soy flour proportion had a rising effect on cohesiveness. Protein level was the dominant component of milk powder and soy flour, so high protein densities in white compound chocolate formulation are associated with high value of cohesiveness. According to Zarić et al. (2015), the presence of soy proteins was found to increase the cohesiveness of chocolate. They also observed substitution of cow milk powder by soy milk powder caused more cohesive texture. Increasing the sesame paste proportion in white chocolate compound formulation caused a decrease in cohesiveness. This could partly be attributed to a rise in vegetable oils resulting in less flexible protein network. Oil as a viscose ingredient in food structure acts as a particle‐space barrier and reduced their contact. The addition of oil was expected to reduce the flexibility of food structure.

The effect of emulsifier on cohesiveness at constant proportion of milk powder (5% w/w) is given in Figure 2b. At low proportion of sesame paste, increasing emulsifier up to a certain value decreased the cohesiveness. Seemingly, emulsifier coats particles and reduces interparticle interactions. This makes the molecules an effective dispersant, inducing product softening effect. At higher amount of emulsifier, cohesiveness intensely increased. At concentrations higher than 1% emulsifier, a two‐layered structure of emulsifier is formed around the particles and increases the particle interaction which results in higher cohesiveness. At high level of sesame paste, effect of emulsifier on cohesiveness was not noticeable.

The TPA adhesive force is defined as the necessary force to overcome attractive forces between surface of chocolate and contacting material (Bryant et al., 1995). Adhesive force had linear (mixture components) × linear (emulsifier) model (Table 2). Table 3 showed that for the model of adhesive force, the linear effect of all the mixture components (A, B, and C) and interactions of B and C with emulsifier (D) were significant (*p* < .05). The effect of mixture components and emulsifier on adhesive force is given in Figure 3a,b. The replacement of milk powder by sesame paste had no substantial effect on the white compound chocolate adhesive force (Figure 3a). Increasing sesame oil did not lead to significant increases in the adhesive force. Decrease in sesame paste and milk powder proportion with simultaneous increase in soy flour proportion leads to a decrease in adhesive force. The lowest and highest values of adhesive force were recorded for samples containing 15% soy flour and 15% milk powder, respectively.

According to Figure 3b, the adhesive force was emulsifier dependent and decreased from 8.1 to 5.2 N as the emulsifier concentration increased from 0% to 2% at low sesame paste proportion (15%w/w).

The *T*_onset_ temperature in DSC thermogram was determined by the intersection of the baseline with the absolute highest tangent of the melting curve at which a specific crystal form started to melt. *T*_onset_ had a linear (mixture components) × linear (emulsifier) model where the linear effect of the mixture components (A, B, and C) and the interactions of C with D (emulsifier) were significant (*p* < .05) to the response (Table 3). The effect of mixture components and emulsifier on *T*_onset_ is given in Figure 4a,b. The amount and composition of fat in chocolate formulation changed the crystallinity, melting properties, and crystal size distribution. Chocolate thermal properties affect the perception of taste and oral sensation (Afoakwa et al., 2008a). White compound chocolate containing 15% w/w milk powder showed the greatest stability with *T*_onset_ temperature occurred at 28.1°C, whereas the earliest *T*_onset_ temperature is seen when white compound chocolate contains 15% w/w soy flour (Figure 4a). Apparently, the reduction in *T*_onset_ in the presence of sesame oil and soybean oil was due to the eutectic effect of these oils with CBS made crystals more unstable in the chocolate compound and began to melt at lower temperatures. Afoakwa et al. (2008a) reported that the increasing fat from 25% to 35% did not change *T*_onset_ significantly. On the contrary, in this study, the increase in sesame oil, especially mixture of sesame oil and soybean oil, resulted in a sharp decrease in the *T*_onset_.

As shown by Figure 4b at higher sesame paste proportions (25% w/w), *T*_onset_ did not change with emulsifier, but at lower proportion of sesame paste (15% w/w) *T*_onset_ decreased along with the increase in emulsifier.

The maximum melting temperature (*T*_m_) is the most important parameter of the DSC thermogram, and at this temperature, melting curve reaches its peak and melting rate is greatest (Aidoo et al., 2015). Regarding *T*_m_, Table 2 showed that the model was linear (mixture components) × linear (emulsifier). Table 3 indicated that linear effect of all mixture components and interaction of CD on *T*_m_ were significant (*p* < .05). Effect of mixture components and emulsifier on *T*_m_ are shown in Figure 5a,b. Increasing soy flour and sesame paste proportions from 0% to 15% w/w and 15% to 30% w/w, respectively, led to a decrease in *T*_m_ (Figure 5a). Effect of soy flour was greater than sesame paste. The reduction in *T*_m_ indicated that white compound chocolate containing soybean oil and sesame oil had a lower thermal resistance.

As shown in Figure 5b, the addition of emulsifiers to sesame oil and soybean oil reduced *T*_m_. Afoakwa et al. (2008a) stated that increasing in lecithin concentration affected the dimensions of chocolate fat crystals and thermal properties. Variation of lecithin concentration did not have significant effect on *T*_m_. In this study, the increase in emulsifier concentration in the presence of sesame oil and soybean oil significantly reduced *T*_m_.

### 3.4 Optimization and validation of response surface methodology results

To target the commercial sample's characteristics, our optimization experiments were designed to maximize some of the textural and thermal properties such as hardness, cohesiveness, *T*_onset_, *T*_m_, and minimize adhesive force. Sesame paste, soy flour, milk powder, and emulsifier were selected in the range of 15%–30%, 0%–15%, 0%–15%, and 0%–2% w/w, respectively. Table 4 illustrates the optimum levels leading to the highest value of hardness (367.07 N), cohesiveness (0.63), *T*_onset_ (28.1°C), and *T*_m_ (33.7°C) with lowest adhesive force (8.46 N). These are as follows: 15.5% w/w sesame paste, 7.5% w/w milk powder, 7% w/w soy flour, and 0% w/w emulsifier. This optimization maximized the desirability function to 0.92. Three additional experiments were conducted to test the adequacy of the models for predicting the optimum response values. Table 5 shows that the predicted values are close to the experimental values. The results prove the efficacy of the models used.

**TABLE 4:** Predicted optimum proportions of mixture component and level of process variable which achieved the overall optimum desirability

Mixture component	Low	High	Optimum
Sesame paste	15	30	15.5
Soy flour	0	15	7
Milk powder	0	15	7.5
Emulsifier	0	2	0

**TABLE 5:** Predicted and experimental values of the responses obtained at optimum conditions

Response	Predicted value	Experimental value
Hardness (N)	367.07	361.2 ± 7.2
Cohesiveness	0.63	0.6 ± 0.043
Adhesive force (N)	8.46	7.98 ± 0.65
*T*_onset_ (°C)	28.1	27.65 ± 1.23
*T*_m_ (°C)	33.7	32.84 ± 1.24

Note

*T*_onset_ = onset temperature; *T*_m_ = maximum temperature.

## 4 CONCLUSION

This study was conducted by applying RSM based on combined mixture‐process design to optimize the textural and thermal properties of a natural novel compound chocolate containing sesame paste and compare the results to a commercial sample. RSM modeling data indicated that the mixture components and the amount of emulsifier significantly changed the textual and thermal properties. At a constant value of emulsifier, the linear effect of mixture components (sesame paste, soy flour, and milk powder) on textural and thermal properties was significant. The textural properties are mainly affected by changes in the proportion of sesame paste and milk powder, while the thermal properties are mostly influenced by sesame paste and soy flour. At higher proportion of sesame paste, emulsifier did not have significant effect on the textural and thermal properties compared with a lower proportion of sesame paste. Increase in emulsifier at low percentage of sesame paste differently changed the textural and thermal properties. The D‐optimal combined mixture‐process design can be used to generate mathematical models for predicting the optimum formulation of white compound chocolate containing sesame paste with specific textural and thermal properties. Numerical optimization determined the optimum formulation to be sesame paste 15.5%, milk powder 7.5%, soy four 7%, and emulsifier 0% w/w. Natural novel white chocolate compound containing sesame paste and enriched with soy flour has functional characteristics and unique aroma, which can motive people of different age groups to consume it as a nutritious snack.

## CONFLICT OF INTEREST

The authors declare that they do not have any conflict of interest.
